# Implication of TRIMalpha and TRIMCyp in interferon-induced anti-retroviral restriction activities

**DOI:** 10.1186/1742-4690-5-59

**Published:** 2008-07-09

**Authors:** Laetitia Carthagena, Mélanie C Parise, Mathieu Ringeard, Mounira K Chelbi-Alix, Uriel Hazan, Sébastien Nisole

**Affiliations:** 1 Institut Cochin, Université Paris Descartes, CNRS (UMR 8104), Département des Maladies Infectieuses, 22 rue Méchain, 75014, Paris, France; 2 INSERM U567, Paris, France; 3 CNRS FRE 2937, Institut André Lwoff, Villejuif, France; 4 Université Paris Diderot-Paris 7, UFR de Biochimie, Paris, France

## Abstract

**Background:**

TRIM5α is a restriction factor that interferes with retroviral infections in a species-specific manner in primate cells. Although TRIM5α is constitutively expressed, its expression has been shown to be up-regulated by type I interferon (IFN). Among primates, a particular case exists in owl monkey cells, which express a fusion protein between TRIM5 and cyclophilin A, TRIMCyp, specifically interfering with HIV-1 infection. No studies have been conducted so far concerning the possible induction of TRIMCyp by IFN. We investigated the consequences of IFN treatment on retroviral restriction in diverse primate cells and evaluated the implication of TRIM5α or TRIMCyp in IFN-induced anti-retroviral activities.

**Results:**

First, we show that human type I IFN can enhance TRIM5α expression in human, African green monkey and macaque cells, as well as TRIMCyp expression in owl monkey cells. In TRIM5α-expressing primate cell lines, type I IFN has little or no effect on HIV-1 infection, whereas it potentates restriction activity against N-MLV in human and African green monkey cells. In contrast, type I IFN treatment of owl monkey cells induces a great enhancement of HIV-1 restriction, as well as a strain-tropism independent restriction of MLV. We were able to demonstrate that TRIM5α is the main mediator of the IFN-induced activity against N-MLV in human and African green monkey cells, whereas TRIMCyp mediates the IFN-induced HIV-1 restriction enhancement in owl monkey cells. In contrast, the type I IFN-induced anti-MLV restriction in owl monkey cells is independent of TRIMCyp expression.

**Conclusion:**

Together, our observations indicate that both TRIM5α and TRIMCyp are implicated in IFN-induced anti-retroviral response in primate cells. Furthermore, we found that type I IFN also induces a TRIMCyp-independent restriction activity specific to MLV in owl monkey cells.

## Background

In response to infections, eukaryotes have evolved a wide variety of defense mechanisms. In addition to classical innate and adaptive immunities, a third mode of immunity specific to retroviral infections has recently been identified and termed "intrinsic immunity" [1]. So far, two classes of cellular proteins that specifically interfere with retroviral infections at the cellular level have been identified. The first class of factors is constituted of cytidine deaminases such as APOBEC3G, which induce lethal hypermutation of retroviral genomes (reviewed in [2,3]). The second class of retroviral restriction factors targets capsid proteins of incoming virions and comprises the murine Fv1 and the primate TRIM5α proteins (reviewed in [2,3]).

**Figure 1:**
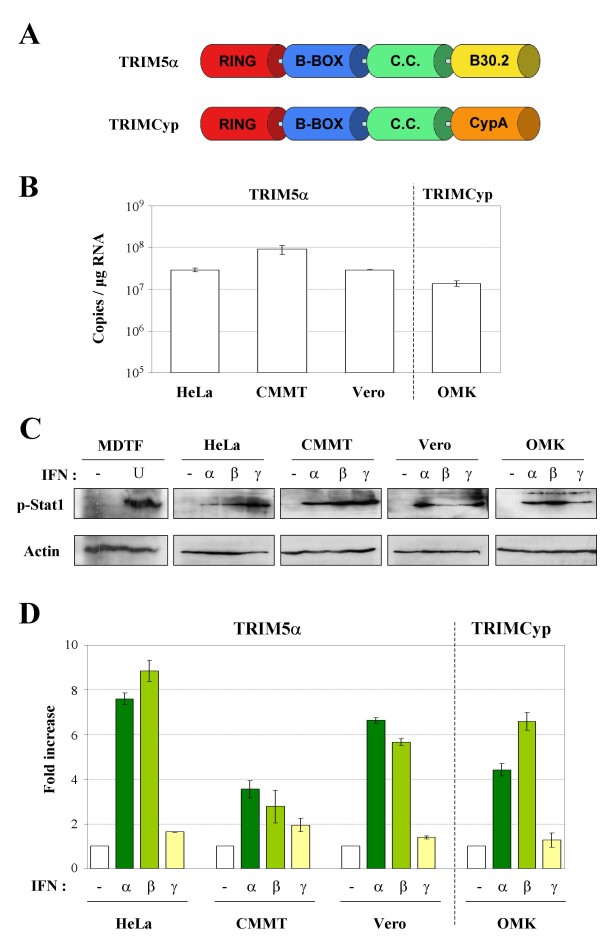
**Up-regulation of TRIM5 expression in primate cells**. **A**. Schematic representation of the domain structure of TRIM5α and TRIMCyp proteins. C.C.: Coiled-Coil. **B**. Comparison of TRIM5α or TRIMCyp constitutive expression in HeLa, CMMT, Vero and OMK cells by quantitative RT-PCR. **C**. MDTF, HeLa, CMMT, Vero or OMK cells were stimulated with universal type I IFN (U), IFN-α, β or γ for 20 min or left untreated (-) and assessed for phosphorylated Stat1 (Tyr 701) and actin by western blot. **D**. Total RNA was extracted after 8 h of treatment with IFN-α, β or γ. TRIM5α (in HeLa, CMMT and Vero cells) or TRIMCyp (in OMK cells) mRNA expression levels were measured by quantitative RT-PCR and normalized to GAPDH mRNA. Results are expressed as fold increase, defined as the ratio of TRIM5 expression in IFN treated/untreated cells. Error bars reflect the SD of duplicate values in a representative experiment.

TRIM5α is responsible for a species-specific post-entry restriction of diverse retroviruses in primate cells [4-8]. TRIM5α is a member of the large family of tripartite motif proteins (TRIM), which is composed of proteins containing a conserved tripartite organization (known as RBCC, for RING, B-BOX, and coiled-coil domains), followed by a C-terminal portion of variable nature (for review, see [9,10]). TRIM5α contains a B30.2/SPRY domain in its C-terminus (Figure 1A), which determines the virus-specific restriction activity of TRIM5α protein from different species [11,12].

TRIM5α protein from Old World monkeys blocks both human immunodeficiency virus type 1 (HIV-1) and N-tropic murine leukemia virus (N-MLV), whereas human TRIM5α only interferes with N-MLV infection. Most TRIM5α variants from New World monkeys restrict simian immunodeficiency virus (SIVmac) but not HIV-1 infection [13], with the notable exception of owl monkey (*Aotus trivigartus*) cells which block HIV-1 but not SIVmac or N-MLV infections. Instead of TRIM5α, owl monkeys express a TRIMCyp fusion protein, generated by retrotransposition of a cyclophilin A (CypA) mRNA within the TRIM5 locus [14,15]. This retrotransposition event led to the expression of a chimeric protein that consists of the RBCC motif of TRIM5 fused to a carboxy-terminal CypA moiety [14,15] (Figure 1A). CypA is a ubiquitously expressed and highly conserved peptidyl prolyl isomerase that can catalyze cis/trans-isomerization of prolyl peptide bonds. This cellular protein has been shown to bind the HIV-1 capsid protein (CA) through a direct interaction between the active site of CypA and an exposed loop within the CA protein, known as the CypA binding loop [16,17]. Capsid proteins from other retroviruses also interact with CypA, such as feline immunodeficiency virus (FIV), SIVcpz or SIVagm, whereas others such as MLV or SIVmac, do not [18,19]. CypA-CA interaction can be disrupted by cyclosporine A (CsA), an immunosuppressive drug that competitively binds to the CypA active site [17,20]. In consequence, since owl monkey TRIMCyp proteins bind CA proteins of incoming retroviruses through their C-terminal CypA domain, restriction can be relieved by CsA treatment [14,15,21,22].

Like many other members of its protein family [23,24], TRIM5α expression has been recently found to be up-regulated by type I interferon (IFN) in human cells, through an IFN-stimulated response element (ISRE) within its promoter [25]. This finding suggested that IFN might influence the retroviral activity of TRIM5α. In the particular case of owl monkey cells, it is not known whether TRIMCyp expression is also enhanced by IFN. We thus tried to determine whether the retroviral restriction profile of different primate cells can be modulated by type I (α, β) and type II (γ) IFNs, and to evaluate the role of TRIM5α and TRIMCyp in IFN-induced anti-retroviral responses.

## Methods

### Cells and viruses

Cell lines of human (*Homo sapiens*, HeLa cells), African green monkey (*Cercopithecus aethiops*, Vero cells), rhesus macaque (*Macaca mulatta*, CMMT cells), and owl monkey (*Aotus trivigartus*, OMK cells) origin were maintained in Dulbecco's modified Eagle's medium containing 10% fetal bovine serum and antibiotics.

Vesicular stomatitis virus glycoprotein (VSV-G)-pseudotyped retroviral vectors were generated by co-transfecting 10-cm plates of 293T cells with 10 μg of pVSV-G, 10 μg of Gag-Pol expression plasmid and 10 μg of green fluorescent protein (GFP)-expressing retroviral vector, using the ProFection calcium phosphate kit (Promega). MLV vectors were made with pCFG2-eYFP and pHIT60 (Mo-MLV, a NB-tropic strain), pCIG3N (N-tropic MLV), or pCIG3B (B-tropic MLV). HIV-1 vectors were prepared with pCSGW (GFP vector) and p8.91 (HIV-1 Gag-Pol). All MLV- and HIV-derived plasmids were kindly provided by J.P. Stoye (National Institute for Medical Research, London, UK). SIVmac vectors were generated with pAd-SIV3+ and GAE-CMV-GFP, which were kindly provided by F.L. Cosset (ENS Lyon, France). The HIV SCA Gag-Pol plasmid was a generous gift from J. Sodroski (Dana Farber Cancer Institute, Boston, MA, USA) and was co-transfected with pCSGW, pVSV-G and pRev (10:10:8:2 ratio) to produce HIV-1 SCA viral particles. All viral stocks were titrated on *Mus dunni *tail fibroblast (MDTF) cells and analyzed by FACS. The multiplicity of infection (MOI) is defined as MOI = -2 ln(1-fp), where fp is the fraction of GFP-positive MDTF cells. HeLa, Vero, CMMT or OMK cells were then transduced with increasing doses of GFP-encoding retroviral vectors and the percentage of transduced GFP-positive cells was determined by FACS 48 h post-transduction [7,8].

### Drug and interferon treatment

Cyclosporin A (CsA, Sigma) was prepared at 1 mM in ethanol and diluted further in culture medium before use. For all experiments, IFN-α, IFN-β or IFN-γ were used at 1000 units/ml (U/ml). Human recombinants IFN-α2 is from Schering-Plough, IFN-β is from Cytotech SA and IFN-γ is from Roussel Uclaf. For treating MDTF cells, we used universal type I IFN (PBL Biomedical Laboratories) at 200 U/ml, which was found to be active on most mammalian cells.

### Quantitative RT-PCR

Total RNA was extracted using RNeasy Mini Kit (Qiagen) and cDNA were prepared using a Oligo(dT) primer and SuperScript III Reverse Transcriptase (Invitrogen). Quantitative PCR were performed in duplicates using 1 μl of cDNA on a Roche LightCycler, using the LightCycler Fast Start DNA Master SYBR Green 1 kit (Roche). Primers were synthesized by Eurogentec. TRIM5α: TTGGATCCTGGGGGTATGTGCTGG (forward) and TGATATTGAAGAATGAGACAGTGCAAG (reverse). GAPDH: GGGAAACTGTGGCGTGAT (forward) and GGAGGAGTGGGTGTCGCTGTT (reverse). CypA: AGTGGTTGGATGGCAAGC (forward) and GATTCTAGGATACTGCGAGCAAA (reverse). TRIMCyp: CAGAAGTCCAACGCTACTGGG (forward) and CTTGCCACCAGTGCCATTATGG (reverse). PCR reactions were carried out with a denaturation step of 10 min at 95°C followed by forty-five cycles of 10 s at 95°C, 5 s at annealing temperature (55°C for cyclophilin A, 60°C for TRIM5α, TRIMCyp and GAPDH) and 20 s amplification at 72°C. Quantifications of cDNAs were determined in reference to a standard curve prepared by amplification of serial dilutions of PCR product or plasmids containing matching sequences. Analyses were performed using the second-derivative-maximum method provided by the LightCycler quantification software, version 3.5 (Roche Diagnostics).

### Western blot analysis

Cells untreated or treated with IFN-α, β or γ at 1000 U/ml (or with 200 U/ml of universal type I IFN in the case of MDTF cells) for 20 min were lysed with lysis buffer (20 mM Tris HCl pH7.5, 400 mM NaCl, 1% Triton, 1 mM EDTA, 50 mM KCl and 5 mM β-Mercaptoethanol). Cells extracts (100 μg) were resolved by sodium dodecyl sulphate polyacrylamide gel electrophoresis (SDS-PAGE) and western blotted using anti-phosphorylated Stat1 (Tyr 701) rabbit antibodies (Santa Cruz Biotechnology, Inc.) or an anti-actin mouse mAb (Calbiochem).

### siRNA

Down-regulation of TRIM5α or TRIMCyp expression by siRNA was performed by transfecting cells with siRNA oligos (Dharmacon) using HiPerFect Transfection Reagent (Qiagen) according to manufacturer's instructions. siRNAs targeting human TRIM5α were H1 (GGUCAUUUGCUGGCUUUGU) and HA2 (GCACUGUCUCAUUCUUCAA). For African green monkey (agm) TRIM5α silencing, we used HA2 and A3 (GCCUUACGAAGUCUGAAAC). In order to silence TRIMCyp expression in OMK cells, we used a siRNA targeting CypA (CypA: GGGUUCCUGCUUUCACAGA). Control siRNA transfections were performed using a luciferase control siRNA (Dharmacon).

### Quantification of reverse transcripts

OMK cells untreated or treated with 1000 U/ml of IFN-β were transduced 24 h later with GFP-expressing retroviral vectors pre-treated with DNase (Roche DNase I RNase-free 200 U/ml, 10 mM MgCl_2_, 1 h at room temperature) and harvested 6 h later. Total DNA was extracted using DNeasy Mini Kit (Qiagen) and quantified by measuring OD at 260 nm. Reverse transcripts were detected by PCR on 100 ng of total DNA using primers to GFP: TACGGCAAGCTGACCCTGAAG (forward) and ACGAACTCCAGCAGGACCATG (reverse).

## Results

### Up-regulation of TRIM5α and TRIMCyp expression by IFNs in primate cells

To investigate whether type I and type II IFNs can enhance TRIM5 (TRIM5α or TRIMCyp) expression in various primate cells, we decided to compare cell lines of human (HeLa), African green monkey (Vero), rhesus macaque (CMMT) and owl monkey (OMK) origin.

First, we compared the constitutive expression of TRIM5α (HeLa, Vero and CMMT cells) or TRIMCyp (OMK cells) transcripts in the different cell lines by quantitative RT-PCR (Figure 1B). We found that OMK cells contain approximately 1.5 × 10^7 ^copies/μg total RNA of TRIMCyp transcripts, whereas HeLa and Vero cells contain 3 × 10^7 ^transcripts encoding TRIM5α. Among the four cell lines tested, CMMT cells express the highest number of TRIM5 transcripts, with a concentration of 9 × 10^7 ^copies of TRIM5α per μg of total RNA.

In order to verify if non-human primate cells are responsive to human IFN treatment, we looked at Stat1 phosphorylation, which is an early post-receptor signaling element of both type I and type II IFN pathways, and can thus serve as a positive-indicator of IFN signaling [26]. For this purpose, CMMT, Vero and OMK cells were treated with 1000 U/ml of human IFN-α, β or γ for 20 min and the activation of Stat1 was estimated by western blotting using anti-phosphorylated Stat1 antibodies. As shown in Figure 1C, although the different cell lines do not equally respond to the three IFNs, both human type I (α, β) and II (γ) IFNs can induce Stat1 phosphorylation in non-human primate cells as well as in human cells (HeLa cells). As a control, the same assay was performed on MDTF cells treated with 200 U/ml of universal type I IFN (Fig 1C), which is active on most mammalian cells. MDTF cells are devoid of any post-entry retroviral restricting activity, since they are Fv1-null [27,28], do not encode a TRIM5 ortholog, like all other mouse cells (reviewed in [29]), and were found to express an inactive APOBEC3G protein[30].

Next, we stimulated the primate cell lines with 1000 U/ml of IFN-α, β or γ for 8 h and determined TRIM5α or TRIMCyp mRNA amounts by quantitative RT-PCR. Results were normalized to GAPDH mRNA levels. Figure 1D shows the ratio of TRIM5α or TRIMCyp expression in IFN treated/untreated cell, which is referred to as "Fold increase". We found that type I IFN enhances TRIM5α mRNA expression with induction folds ranging from 3.5 (CMMT) to 7.6 (HeLa) for IFN-α, and from 2.8 (CMMT) to 8.8 (HeLa) for IFN-β. In contrast, IFN-γ treatment does not significantly affect TRIM5α expression in any cell line, with fold induction values ranging from 1.4 (Vero) to 1.9 (CMMT). These results are consistent with previous observations made in human cells [25]. In OMK cells, stimulation by type I IFN leads to a 4.4 (IFN-α) to 6.6 (IFN-β) fold increase of TRIMCyp mRNA expression, whereas type II IFN does not have any significant effect (around 1.3 fold increase).

### Effect of IFNs on retroviral restriction in primate cells

**Figure 2:**
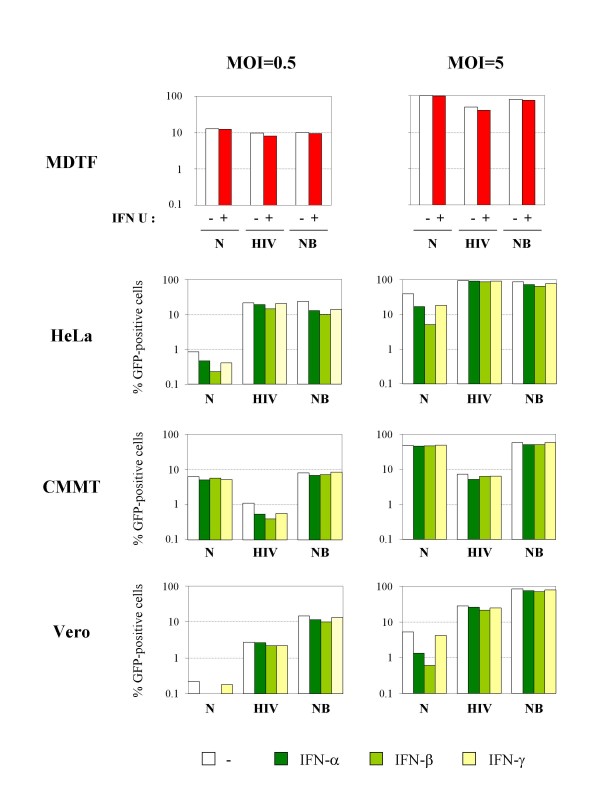
**Modulation of anti-retroviral activity in primate cells by IFNs**. MDTF, HeLa, CMMT, Vero or OMK cells were stimulated with universal type I IFN (U), IFN-α, β or γ or left untreated (-), and transduced 24 h later with VSV-pseudotyped GFP-expressing N-MLV (N), HIV-1 (HIV) or NB-MLV (NB) at low (MOI = 0.5) or high (MOI = 5) multiplicity of infection (as determined on MDTF cells). The percentage of transduced (GFP-positive) cells was determined by FACS 48 h post-transduction.

We next examined whether the increase in TRIM5α or TRIMCyp mRNA following type I IFN treatment can influence the cell permissivity to retroviral infections. For this purpose, VSV-G-pseudotyped, GFP-encoding retroviral vectors derived from HIV-1, N-MLV and NB-MLV were prepared by transfection of 293T cells (see methods) and titrated on MDTF cells. HeLa, CMMT, Vero or OMK cells were stimulated with IFN-α, β or γ and challenged with increasing doses of GFP-encoding retroviral vectors 24 h later. MDTF cells were stimulated with universal type I IFN and challenged with the same dose of virus. The percentage of transduced GFP-positive cells was determined by FACS 48 h post-transduction. Figure 2 shows the results obtained when MDTF, HeLa, CMMT and Vero cells were challenged with either a small (MOI of 0.5 on MDTF cells) or a large (MOI of 5 on MDTF cells) amount of virus.

In the absence of IFN treatment, N-MLV is restricted in HeLa and Vero cells, whereas HIV-1 is blocked in CMMT and Vero cells. As expected, NB-MLV infection is not affected in any cell line. Furthermore, IFN-treatment of MDTF cells, which are devoid of any restriction factor, does not affect their capacity to be transduced by any retroviral vector. We did not observe any dramatic effect on retroviral restriction levels in IFN-treated primate cells at this low multiplicity of infection. IFN-β was found to be the most effective in enhancing TRIM5α-mediated restriction, in accordance with the results of expression enhancement. Following IFN-β treatment, we observed a low but reproducible enhancement of N-MLV restriction in HeLa and Vero cells, as well as a higher restriction towards HIV-1 infection in CMMT cells. This moderate effect of IFN at low MOI is probably due to the fact that the constitutive amount of TRIM5α in the cells is sufficient to restrict a small amount of virus, whereas at higher MOI, it can be predicted that an increased expression of TRIM5α would prevent its saturation by incoming viruses, thus enhancing restriction. Indeed, when a higher dose of virus was used (MOI of 5 on MDTF cells), we observed a much higher restriction efficiency of N-MLV in HeLa and Vero cells treated with type I IFN (Figure 2). In contrast, no significant increase of HIV-1 restriction can be observed in Vero or CMMT cells.

Together, our observations confirm that type I IFN can modulate retroviral restriction in diverse primate cells. However, we did not observe a direct and systematic correlation between the levels of up-regulation of TRIM5α expression and the enhancement of restriction activity. Indeed, the IFN-induced reduction of infectivity of TRIM5α-sensitive viruses seems to be dependent of diverse parameters, such as the multiplicity of infection and the basal restriction activity of cells against a given virus.

**Figure 3:**
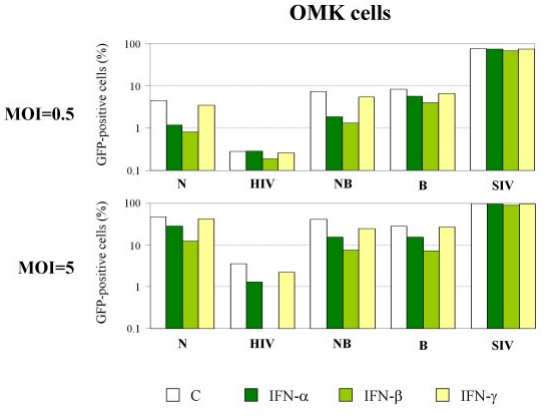
**IFN-induced anti-retroviral activities in primate cells**. OMK cells were treated with human IFN-α, β or γ and transduced 24 h later with VSV-pseudotyped GFP-expressing N-MLV (N), HIV-1 (HIV), NB-MLV (NB), B-MLV (B) or SIVmac (SIV) at low (MOI = 0.5) or high (MOI = 5) multiplicity of infection. The percentage of transduced cells was determined by FACS 48 h post-transduction.

In OMK cells, treatment with type I IFN was found to have a greater influence on retroviral permissivity compared to other primate cell lines (Figure 3). IFN-β treatment, in particular, induces a 3-fold decrease of HIV-1 infectivity at low multiplicity of infection (MOI of 0.5), which rises to 26-fold at high MOI (MOI of 5). More surprisingly, stimulation with type I IFN also led to a reduced susceptibility of OMK cells to NB-MLV and N-MLV infection, although TRIMCyp is known to be inefficient to restrict MLV strains, since MLV CA proteins do not interact with CypA. Indeed, we found that IFN-β induces a 3.9 (at high MOI) to 5.6 (at low MOI) fold increase of restriction towards N-MLV and a 5.5 (at high MOI) to 5.6 (at low MOI) fold increase of anti-NB-MLV restriction activity in OMK cells (Figure 3 and Table 1). In order to determine whether IFN initiates a wide and unspecific antiviral response in OMK cells, we tested two other viruses which are known to be insensitive to TRIMCyp-mediated restriction, B-MLV and SIVmac (Figure 3) [17,18,22]. Interestingly, B-MLV permissivity is also affected by IFN treatment, whereas OMK cells remain fully permissive to SIVmac. The fact that SIVmac is unaffected by this IFN-induced block proves that the loss of infectivity we observed with MLV is not the consequence of an increased cell mortality due to IFN treatment. Since IFN-β gave more contrasted results than IFN-α, we decided to pursue our study with IFN-β only. Table 1 summarizes the effect of IFN-β on retroviral restriction in all tested primate cell lines. Results are expressed as fold restriction, which represents the ratio of untreated to IFN-treated cells to be transduced by the GFP-encoding retroviral vectors. As shown, IFN-β significantly increases (fold restriction > 2) the restriction of N-MLV in HeLa and Vero cells, whereas it induces a wide-spectrum anti-retroviral activity in OMK cells affecting HIV-1, NB-MLV, N-MLV and B-MLV but not SIVmac.

**Table 1:** Effect of IFN-β treatment on retroviral restriction.

	**FOLD RESTRICTION**									
	
	**HIV-1**		**N-MLV**		**NB-MLV**		**B-MLV**		**SIVmac**	
MOI	0.5	5	0.5	5	0.5	5	0.5	5	0.5	5
**MDTF**	1.2	1.0	1.0	1.1	1.1	1.0	1.1	1.1	1.1	1.0
**HeLa**	1.5	1.1	**3.7**	**7.6**	1.9	1.4	nt	nt	nt	nt
**CMMT**	**2.8**	1.2	1.1	1.0	1.1	1.1	nt	nt	nt	nt
**Vero**	1.2	1.3	**4.4**	**8.8**	1.5	1.2	nt	nt	nt	nt
**OMK**	**2.9**	**26.1**	**5.6**	**3.9**	**5.6**	**5.5**	**2.1**	**3.9**	1.1	1.1

HeLa, CMMT, Vero or OMK cells were stimulated with IFN-β and transduced 24 h later at a MOI of 0.5 or 5 (as determined on MDTF cells) with HIV-1, N-MLV, NB-MLV, B-MLV or SIVmac, as indicated. The same experiment was performed in parallel on MDTF cells treated or not with universal type I IFN. Fold restriction represents the ratio of untreated to IFN-treated cells to be transduced by the GFP-encoding retroviral vectors. A significant enhancement of retroviral restriction is defined as a ratio > 2 (in bold). Data are from a typical experiment representative of three independent experiments. nt: not tested.

Therefore, our observations suggest that OMK cells express an IFN-inducible anti-retroviral protein other than TRIMCyp, which can interfere specifically with certain retroviruses.

### The IFN-induced anti-retroviral activity in HeLa and Vero cells is TRIM5α-dependent

**Figure 4:**
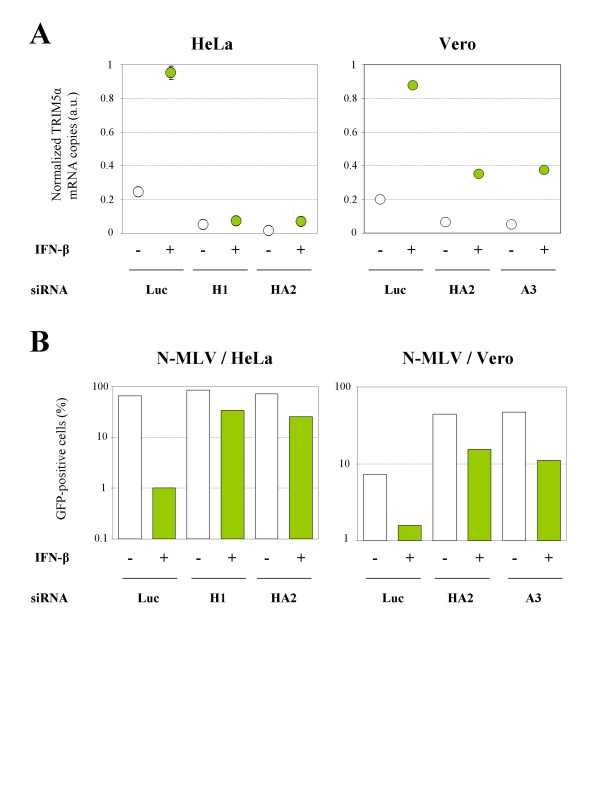
**TRIM5α is the main mediator of the IFN-induced N-MLV restriction**. **A**. HeLa and Vero cells were transfected with a siRNA targeting luciferase (Luc) or TRIM5α (siRNA H1, HA2 or A3), as indicated, and stimulated 24 h later with 1000 U/ml of IFN-β for 8 h. Total RNA was extracted and the levels of TRIM5α mRNA were determined by quantitative RT-PCR and normalized to GAPDH. The mean ± SD of duplicates is shown. **B**. HeLa or Vero cells were transfected with siRNA targeting luciferase (Luc), TRIM5α_hu _(H1 or HA2) or TRIM5α_agm _(HA2 or A3), as indicated. The next day, cells were stimulated with 1000 U/ml of IFN-β for 8 h and challenged with a GFP-expressing N-MLV vector. The percentage of GFP-positive cells was determined by FACS 48 h post-transduction. Data are from a typical experiment representative of three independent experiments.

In order to verify whether the increased restriction activity towards N-MLV infection in HeLa and Vero cells was due to the IFN-induced TRIM5α, cells were first transfected with a siRNA targeting TRIM5α, treated with IFN-β 24 h later, and finally challenged the next day with N-MLV at a MOI of 5 (as determined on MDTF cells). Three siRNA were used, which silence human (H1 and HA2) or African green monkey (HA2 and A3) TRIM5α expression (Figure 4A). A siRNA targeting luciferase (Luc) was used as a control. As shown in Figure 4B, the IFN-induced enhancement of N-MLV restriction in HeLa and Vero cells is almost completely lost when TRIM5α expression is down-regulated by either of the two siRNA, thus suggesting that TRIM5α is the main mediator of the IFN-induced N-MLV restriction. In contrast, no effect on B- or NB-MLV infectivity can be observed following TRIM5α silencing in HeLa or Vero cells, as expected (not shown).

### The IFN-induced anti-MLV activity in OMK cells is TRIMCyp-independent

**Figure 5:**
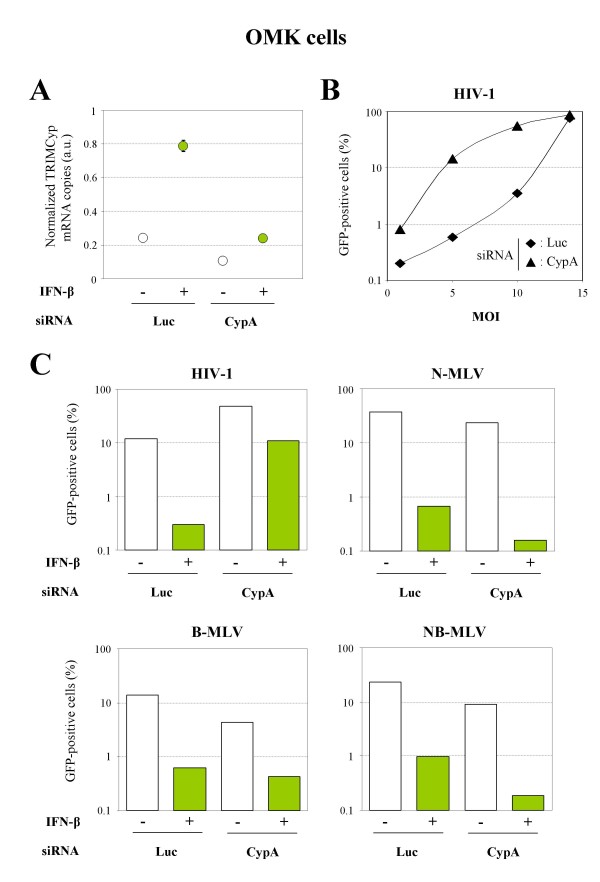
**The IFN-induced MLV restriction activity in OMK cells is independent of TRIMCyp**. **A**. OMK cells were transfected with a siRNA targeting luciferase (Luc) or TRIMCyp (as well as CypA), as indicated, and stimulated 24 h later with 1000 U/ml of IFN-β for 8 h. Total RNA was extracted and the levels of TRIMCyp mRNA were determined by quantitative RT-PCR and normalized to GAPDH. The mean ± SD of duplicates is shown. **B**. OMK cells were transfected with anti-Luc (diamonds) or anti-TRIMCyp (triangles) siRNA and transduced 48 h later with increasing doses of HIV-1. The percentage of GFP-positive cells was determined by FACS 48 h post-transduction. **C**. Same experiment as in panel B, except that cells were challenged with HIV-1, N-MLV, B-MLV or NB-MLV (at a MOI of 5), following siRNA and IFN-β treatments. The percentage of GFP-positive cells was determined by FACS 48 h post-transduction. Data are from a typical experiment representative of three independent experiments.

We used the same strategy in order to confirm the existence of an IFN-induced TRIMCyp-independent retroviral restriction activity in OMK cells. For this purpose, we used a siRNA targeting CypA as well as the CypA C-terminal portion of TRIMCyp. The anti-Luc siRNA was used as control. Figure 5A shows the quantification of TRIMCyp expression in siRNA-transfected OMK cells by quantitative RT-PCR. As expected, OMK cells transfected with the TRIMCyp siRNA lost their capacity to restrict HIV-1, as compared to cells transfected with the Luc siRNA (Figure 5B). Next, we tested the effect of TRIMCyp silencing on retroviral restriction following IFN-β stimulation. One day after siRNA transfection, OMK cells were stimulated with 1000 U/ml of IFN-β and challenged with one of the four retroviruses found to be restricted: N-MLV, B-MLV, NB-MLV or HIV-1. All retroviral vectors were used at the same titer, corresponding to the viral dose that gives a MOI of 5 on MDTF cells. As shown in Figure 5C, the IFN-β-induced restriction of N-, B- and NB-MLV is not affected by TRIMCyp silencing, thus demonstrating that another mediator is involved in this anti-retroviral activity. In the case of HIV-1, the extinction of TRIMCyp expression prevents the IFN-induced enhancement of viral restriction, confirming the fact that TRIMCyp is the main mediator of the IFN-induced anti-HIV-1 activity in OMK cells.

**Figure 6:**
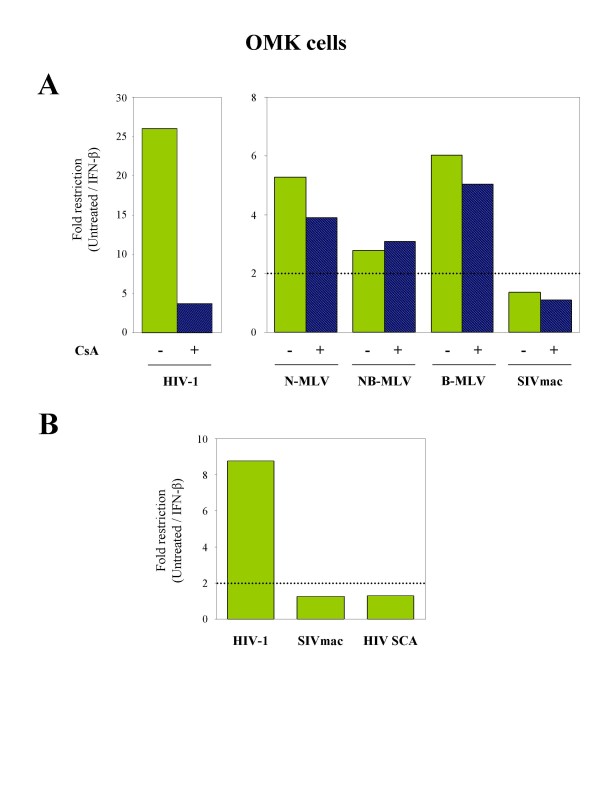
**The IFN-induced enhancement of anti-HIV-1 restriction in OMK cells is due to TRIMCyp**. **A**. OMK cells were induced with IFN-β and challenged 24 h later with GFP-expressing HIV-1, N-MLV, B-MLV or NB-MLV at a MOI of 5. CsA (5 μM) was added 2 h before transduction. Percentage of transduced cells was determined by FACS 48 h post-transduction. Fold restriction represents the ratio of untreated to IFN-β-treated cells to be transduced by the GFP-encoding retroviral vectors. Results are representative of two independent experiments with comparable results. A significant enhancement of retroviral restriction is defined as a ratio > 2. **B**. OMK cells were stimulated with IFN-β and transduced 24 h later with VSV-pseudotyped GFP-expressing HIV-1, SIVmac or HIV-1 SCA vectors at a MOI of 1. The percentage of transduced cells was determined by FACS 48 h post-transduction. Fold restriction represents the ratio of untreated/IFN-β-treated cells to be transduced. As in panel A, a significant enhancement of retroviral restriction is defined as a ratio > 2.

To further address the question of the involvement of TRIMCyp in the IFN-induced anti-retroviral activities, we examined the effect of IFN-β stimulation on retroviral transduction in the presence of CsA, which is known to relieve the TRIMCyp-mediated restriction in OMK cells [14,15,21,22]. OMK cells were stimulated with 1000 U/ml of IFN-β and challenged with retroviral vectors 24 h later in the presence of CsA. We found that IFN-induced restriction of HIV-1 was partially relieved by CsA, thus confirming that TRIMCyp is the main mediator of the IFN-induced anti-HIV restriction in OMK cells (Figure 6A). In contrast, CsA treatment had no effect on the IFN-induced activity against N-, B- and NB-MLV, thus confirming that TRIMCyp is not responsible for the IFN-induced block of MLV infection.

This was further confirmed by comparing the restriction profile of HIV-1, SIVmac and HIV-1 containing the CA protein of SIVmac (HIV SCA) following IFN-β stimulation of OMK cells. As shown in Figure 6B, the HIV SCA chimera virus is as insensitive to IFN-β treatment as SIVmac, compared to HIV-1.

### The IFN-induced block in OMK cells targets an early step of MLV replication

Since IFNs can induce several antiviral mediators acting at different stages of viral replication, we next examined which step of the viral cycle is inhibited in response to IFN treatment of OMK cells.

**Figure 7:**
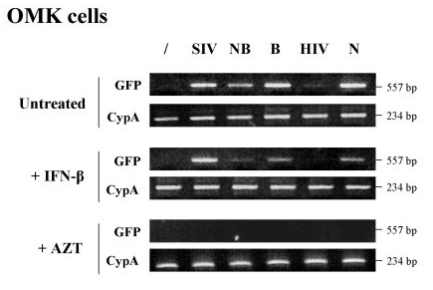
**The IFN-induced block occurs early in the MLV replication cycle**. OMK cells were treated or not with 1000 U/ml of IFN-β and challenged 24 h later with DNase-treated GFP-expressing SIVmac (SIV), HIV-1 (HIV), N-, B-, or NB-tropic MLV vectors. The same experiment was performed in parallel in the presence of AZT at 5 μM during infections. Total DNA was extracted 6 h post-transduction and the amount of reverse transcripts was estimated by PCR using GFP primers. A PCR on CypA was also performed as a control.

First, we challenged naive or IFN-β-treated OMK cells with SIVmac, NB-MLV, B-MLV, HIV-1 or N-MLV and extracted total DNA 6 h post-transduction. Reverse transcripts were detected in cell extracts by PCR using GFP primers. In accord with the mode of action of TRIMCyp, we observed an inhibition of viral DNA synthesis in the case of HIV-1 infection, which is even more pronounced when cells are pretreated with IFN-β (Figure 7). In contrast, the amount of SIVmac reverse transcripts is comparable between naive and IFN-stimulated cells, as expected. Interestingly, the amount of reverse transcripts significantly decreases in cells treated with IFN-β following infection with NB-, B- or N-tropic MLV strains. We concluded from this experiment that IFN-β treatment interferes with an early step of the MLV replication cycle, prior to or during reverse transcription.

## Discussion

In this study, we have shown that type I (α and β) IFN increases TRIM5α expression in human, African green monkey and rhesus macaque cell lines, as well as TRIMCyp expression in owl monkey cells. A previous study reported that human TRIM5α expression can be directly up-regulated by IFN-β through a putative interferon-stimulated response element (ISRE) within its promoter [25]. Our data are in agreement with the results published by Asaoka *et al*. [25], as we observed in HeLa cells an 8- to 9-fold increase of TRIM5α expression with IFN-β and no effect with IFN-γ. In contrast, we found that IFN-α was almost as efficient as IFN-β in stimulating human TRIM5α expression, in agreement with another recent study [31]. It should be noted that the TRIM5α expression increase in CMMT cells is not as strong as in the other primate cells, with a 3- to 3.5-fold increase following stimulation by IFN-α or IFN-β, respectively.

We found that this type I IFN enhancement of TRIM5α expression can influence cell permissivity towards retroviral infections in diverse primate cells. In most cases, we observed a greater effect of IFN stimulation when high concentrations of virus were used. This probably reflects the fact that the constitutive amount of TRIM5α is sufficient to block a small number of incoming viruses, whereas at high MOI, an enhancement of TRIM5α intracellular concentration following IFN treatment facilitates restriction by preventing TRIM5α saturation.

In human cells, IFN-β treatment increases TRIM5α expression by approximately 9-fold, and leads to a 7- to 8-fold reduction of N-MLV permissivity. In contrast, IFN treatment does not affect the permissivity of TRIM5α_hu_-insensitive viruses, such as NB-MLV or HIV-1. These first observations confirm a previous report showing that IFN-α treatment of human cells both increases the level of TRIM5α mRNA and potentates N-MLV restriction by approximately 5-fold [31].

In the case of CMMT and Vero cells, TRIM5α expression is also up-regulated following type I IFN treatment, but in these cases, the increase of expression does not strictly correlate with a decreased infectivity of TRIM5α-sensitive viruses.

In rhesus macaque cells, at low MOI, we only observed a moderate decrease of HIV-1 infectivity (2- to 3-fold reduction), in agreement with Sakuma et al. [31]. In contrast, no effect on N-MLV infection can be observed. Surprisingly, when a higher MOI was used, IFN-treated CMMT cells showed no difference of susceptibility to either N-MLV or HIV-1 infection, compared to untreated cells. This relative insensitivity of CMMT to IFN treatment probably reflects the fact that these cells constitutively express high amount of TRIM5α (Figure 1B) compared to the other primate cell lines. Furthermore, this high constitutive expression is conjugated to a moderate enhancement of TRIM5α expression in response to IFN (Figure 1D).

In Vero cells, we detected no effect of type I or type II IFNs at low MOI, whereas at high MOI, we observed an enhancement of N-MLV restriction following induction by type I IFN only. Unlike N-MLV, HIV-1 infectivity was not found to be significantly affected by the IFN-induced up-regulation of TRIM5α expression. We believe that this apparent discrepancy may be explained by the basal restriction phenotype in Vero cells. Indeed, at constitutive levels of TRIM5α expression, Vero cells restrict N-MLV infection much more efficiently than HIV-1, suggesting that their TRIM5α proteins recognize N-MLV CA better than HIV-1 CA. Our observations suggest that IFN stimulation can potentate restriction towards highly sensitive viruses, but is not sufficient to enhance the restriction activity towards less sensitive ones.

Importantly, we were able to demonstrate that the IFN-induced restriction increase towards N-MLV observed in human and African green monkey cells was mainly due to the enhancement of TRIM5α expression, demonstrating that TRIM5α is the main mediator of the anti-retroviral activity of type I IFN in these cells.

Several studies have demonstrated that IFN-α can interfere with early and late steps of HIV-1 replication, *in vitro *[32-35]. Although we have only tested the effect of IFN on HeLa cells rather than primary blood cells, our results are not in favor of a direct implication of TRIM5α in the early anti-HIV-1 block induced by IFN in human cells. Indeed, up-regulation of TRIM5α expression by type I IFN was not found to correlate with a significant decrease in HIV-1 permissivity. In this context, APOBEC3G, another cellular IFN-induced anti-retroviral factor, is more likely to play a role as a mediator of the early IFN-α-mediated anti-HIV block in human cells [36], whereas the possible involvement of TRIM5α in the late steps remains to be addressed [31].

The second part of our study focused on the characterization of IFN-induced modulation of retroviral infection susceptibility in owl monkey cells. Cells derived from this New World monkey species express a TRIMCyp fusion protein which allows them to specifically interfere with viruses whose capsid can bind CypA, such as HIV-1, FIV and SIVagm [18,22]. In contrast, MLV and SIVmac capsid proteins do not bind CypA and these viruses are in consequence insensitive to TRIMCyp restriction.

First, we observed a 3 (at a MOI of 0.5) to 26-fold (at a MOI of 5) reduction of HIV-1 permissivity following stimulation of OMK cells with type I IFN, which can be attributed almost entirely to the IFN-induced up-regulation of TRIMCyp expression.

Surprisingly, in addition to this strong enhancement of HIV-1 restriction following type I IFN stimulation of OMK cells, we also observed a significant and reproducible restriction of MLV. This MLV block is independent of the strain tropism, since N-, B- and NB-tropic MLV strains were found to be sensitive. Many mammalian cells are able to restrict MLV, but in all cases, the block only affects N-tropic strains [37], with the exception of murine NIH-3T3 cells which express a B-tropic MLV-specific restriction factor, known as Fv1^n ^(reviewed in [38,39]). We were able to demonstrate that this IFN-induced anti-MLV activity is independent of TRIMCyp expression and occurs early during the MLV replication cycle, before or during the reverse transcription step. In contrast to MLV, we found that SIVmac was not affected by IFN-treatment in OMK cells. Even though the effect of IFN-treatment in OMK cells has never been investigated, this observation was unexpected. Indeed, in human cells, IFN-treatment was found to block an early step of SIVmac replication, between viral entry and reverse transcription [40,41]. Furthermore, IFNs are known to activate multiple antiviral proteins which induce a wide-spectrum antiviral response. Thus, our results suggest the absence of IFN-induced antiviral response against SIVmac in OMK cells.

**Figure 8:**
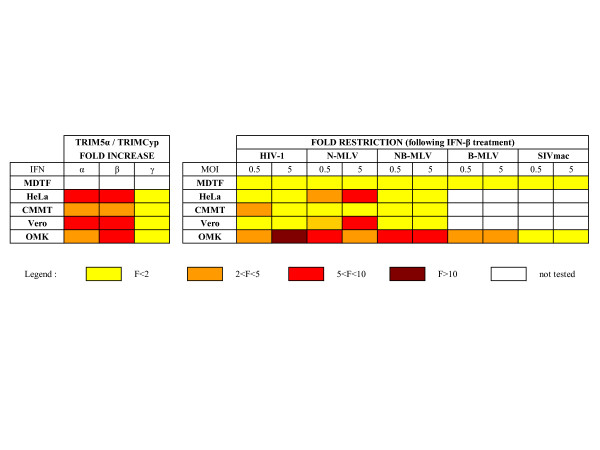
**Effects of IFNs on TRIM5 expression and retroviral restriction in primate cells**. Left: Effect of IFNs on TRIM5α (HeLa, CMMT and Vero) and TRIMCyp (OMK) mRNA expression, expressed as fold increase (IFN-treated/untreated cells). Right: Effect of IFN-β treatment on retroviral restriction, expressed as fold restriction (untreated/IFN-treated cells).

Our main results on the effect of IFNs on TRIM5α or TRIMCyp expression and on retroviral restriction in primate cells are summarized in a heat-map representation (Figure 8).

Several cellular proteins have been identified as mediators of the antiviral activity of IFNs, such as Protein Kinase RNA-dependent, 2'5' oligoadenylate synthetase/RNase L, and certain Mx proteins (reviewed in [42]). Interestingly, in addition to these well characterized IFN-induced antiviral mediators, some other proteins belonging to the TRIM protein family have also been involved in IFN-induced antiviral defense, such as TRIM5α [25,31], PML/TRIM19 (for review, see [43,44]), TRIM22 [45,46] or TRIM25 [47]. These observations suggest that the entire TRIM family may constitute a family of antiviral factors implicated in the IFN-induced intracellular innate immunity [9]. In this respect, a systematic study of the expression of mouse TRIM genes in immune cells in response to various stimuli, including IFN treatment and viral infection, provides new insights into the implication of the TRIM family in antiviral defense[48]. The number of TRIM proteins up-regulated by IFN and/or implicated in antiviral resistance raises the possibility of the involvement of a TRIM protein other than TRIMCyp in the IFN-induced anti-MLV restriction we observed in OMK cells. Apart from TRIMCyp, the only TRIM protein that has been characterized so far in OMK cells is TRIM1 [8]. However, since it was found to interfere specifically with N- but not B-tropic strains of MLV, this protein is unlikely to play a role in the observed phenotype. Whether it belongs to the TRIM protein family or not, the identification of the mediator(s) of the IFN-induced anti-MLV restriction in owl monkey cells will need further investigation.

## Competing interests

The authors declare that they have no competing interests.

## Authors' contributions

LC carried out most of the experimental work and contributed to the analysis of data and the writing of the manuscript. MCP and MR contributed to the experiments. MCA and UH participated in the design of the study and helped to draft the manuscript. SN conceived of the study, participated in its design and coordination and wrote the manuscript. All authors read and approved the final manuscript.
